# Linking F-box protein 7 and parkin to neuronal degeneration in Parkinson’s disease (PD)

**DOI:** 10.1186/s13041-016-0218-2

**Published:** 2016-04-18

**Authors:** Zhi Dong Zhou, Sushmitha Sathiyamoorthy, Dario C. Angeles, Eng King Tan

**Affiliations:** National Neuroscience Institute of Singapore, 11 Jalan Tan Tock Seng, Singapore, 308433 Singapore; Department of Neurology, Singapore General Hospital, Outram Road, Singapore, 169608 Singapore; Signature Research Program in Neuroscience and Behavioural Disorders, Duke-NUS Graduate Medical School Singapore, 8 College Road, Singapore, 169857 Singapore

**Keywords:** FBXO7, Mitochondria, Mitophagy, Parkin, Parkinson’s disease, Protein aggregation, Proteotoxicity, Ubiquitin proteasome system

## Abstract

Mutations of F-box protein 7 (FBXO7) and Parkin, two proteins in ubiquitin-proteasome system (UPS), are both implicated in pathogenesis of dopamine (DA) neuron degeneration in Parkinson’s disease (PD). Parkin is a HECT/RING hybrid ligase that physically receives ubiquitin on its catalytic centre and passes ubiquitin onto its substrates, whereas FBXO7 is an adaptor protein in Skp-Cullin-F-box (SCF) SCF^FBXO7^ ubiquitin E3 ligase complex to recognize substrates and mediate substrates ubiquitination by SCF^FBXO7^ E3 ligase. Here, we discuss the overlapping pathophysiologic mechanisms and clinical features linking Parkin and FBXO7 with autosomal recessive PD. Both proteins play an important role in neuroprotective mitophagy to clear away impaired mitochondria. Parkin can be recruited to impaired mitochondria whereas cellular stress can promote FBXO7 mitochondrial translocation. PD-linked FBXO7 can recruit Parkin into damaged mitochondria and facilitate its aggregation. WT FBXO7, but not PD-linked FBXO7 mutants can rescue DA neuron degeneration in Parkin null *Drosophila*. A better understanding of the common pathophysiologic mechanisms of these two proteins could unravel specific pathways for targeted therapy in PD.

## Background

Parkinson’s disease (PD) is one of the most common neurodegenerative disorder characterized by chronic and progressive loss of dopaminergic neurons in substansia nigra pars compacta (SN). PD can affect about 2 % of the population above 65 years of age [1–3]. PD symptoms include rigidity, postural instability, tremor at rest and slowness or absence of voluntary movement, and even neuropsychiatric symptoms [4, 5]. The pathological hallmarks of PD include progressive degeneration of dopamine (DA) neurons in SN [5, 6] as well as accumulation of α-synuclein (α-syn) positive Lewy bodies in afflicted brain regions [7–9]. Although various hypotheses, including oxidative stress [10], mitochondrial dysfunction [11, 12], impairment of the ubiquitin proteasome system (UPS) and defects in autophagy process [1, 4, 11], have been proposed to be implicated in progressive loss of DA neurons in PD, the exact mechanisms accounting for DA neuron demise in PD still remains to be elucidated [13].

Though most PD cases are late onset and may be classified as sporadic PD (SPD), gene mutations or variations can lead to early onset inherited familial PD (FPD) [3, 14]. Accumulative evidence from studies on FPD have significantly deepened our understanding of PD pathogenesis [15]. The recessive mutations in Parkin gene (PARK2) are associated with classic Levodopa responsive FPD [16]. However recessive gene mutations of FBXO7 (PARK15) are associated with juvenile onset Parkinsonism frequently accompanied with atypical features including dementia, dystonia, hyperreflexia and pyramidal signs [17, 18]. Here, we discuss the overlapping pathophysiologic mechanisms and clinical features linking Parkin and FBXO7 with autosomal recessive PD.

### UPS dysfunction, proteotoxicity and PD pathogenesis

There is increasing evidence to suggest that dysfunction of UPS plays a major role in PD pathogenesis. The function of UPS is to target and degrade unneeded or damaged proteins by proteolysis, a chemical reaction that breaks peptide bonds. The UPS processes involve targeted conjugation of multiple ubiquitin molecules to protein substrates and subsequent degradation of polyubiquitin tagged proteins by proteasome [19]. The process will finally yield peptides of about seven to eight amino acids long, which can be further degraded into shorter amino acid fragments for new proteins synthesis [20]. It was reported that proteasome inhibition induced UPS impairment can result in accumulation of misfolded proteins and deleterious protein aggregates, contributing to neuronal dysfunction and demise [21]. The accumulation of misfolded and aggregated proteins induced toxicity is termed as proteotoxicity, which has been found to be implicated in pathogenesis of various human disorders, including carcinogenesis, neurodegenerative diseases, aging process, cardiovascular disorders, diabetes and many other human diseases [22–25]. Recent findings indicate that PD-linked FBXO7 mutations aggravate aggregation of FBXO7 proteins in mitochondria contributing to FBXO7-linked mitochondria proteotoxicity, which is implicated in FBXO7 mutation induced DA neuron degeneration in PD [26].

Impairment of UPS in PD pathogenesis was established with mutations of Parkin, a HECT/RING hybrid ubiquitin E3 ligase [27, 28], and other genetic forms of PD [20, 29]. The impairment of UPS functions is also implicated in DA neuron degeneration in SPD. The DA in DA neurons can be an endogenous deleterious factor to impair UPS function via irreversible conjugation of protein cysteine residues by highly reactive DA oxidation derived DA quinones [30, 31]. DA quinone is reported to covalently modifies Parkin in living dopaminergic cells, leading to Parkin insolubility and inactivation of its E3 ubiquitin ligase function [32]. These findings show vulnerabilities of Parkin and UPS to reactive DA quinone, suggesting a mechanism for DA quinone induced progressive UPS impairment and Parkin inhibition in dopaminergic neurons during aging and SPD [30–32].

### FBXO7 and FBXO7-linked PARK15

**Fig. 1:**
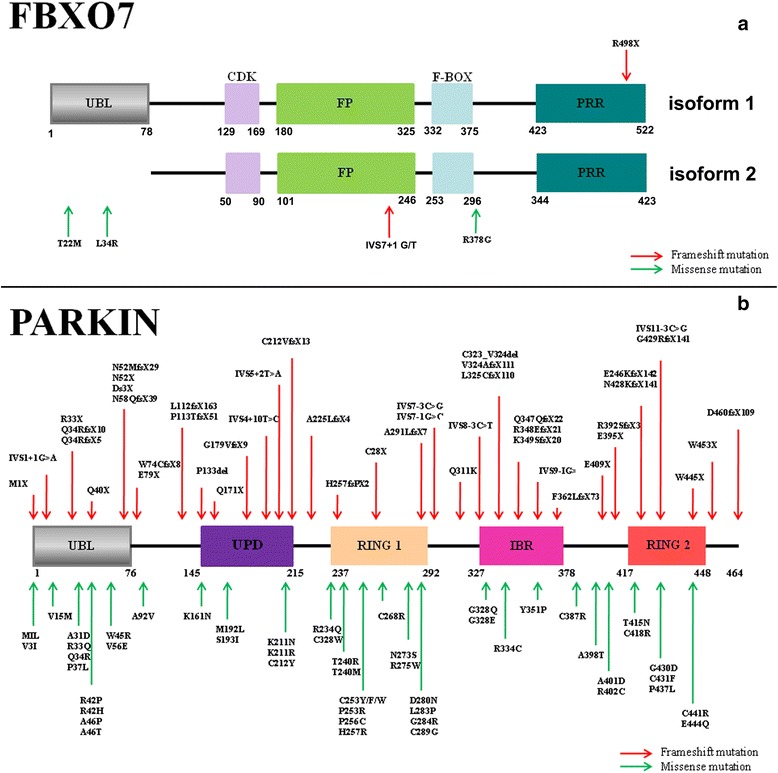
Molecular structure and PD-linked generic variations of FBXO7 and Parkin. The molecular structures of FBXO7 (**a**) and Parkin (**b**) together with indications of PD-linked mutations are respectively illustrated. The detailed sites of PD-linked generic variations of frameshift or missense mutations of FBXO7 and Parkin are pointed with red or green colour arrows respectively

The FBXO7 R378G mutation was first reported to be linked to early-onset Parkinsonian (PARK15) in 2008 [33]. In 2009 R498X and T22M FBXO7 mutations were subsequently indentified to induce autosomal recessive, early-onset Parkinsonian-pyramidal syndrome [17, 33]. The PARK15 patients presented with Babinski signs and spastic weakness [18, 34, 35]. Parkinsonism with resting tremor, bradykinesia and postural instability developed in later stages of some PARK15 patients [36]. Recently a new FBXO7 L34R mutation causing typical L-dopa-responsive Parkinsonism was reported in a Turkish family [37]. The L34R mutation is similar to T22M mutation, which is predicted to affect the UBL domain of FBXO7 isoform 1 protein and is associated with disturbed nuclear localization of FBXO7 protein [37]. Another heterozygous mutation (R481C) of FBXO7 was reported in an Italian family [38, 39]. As these patients also carried other genetic variants related to PD, the pathogenicity of R481C FBXO7 mutant still remains to be clarified [17, 18]. However a potential protective Y52C FBXO7 polymorphism is recently identified [40]. The molecular structure of FBXO7 protein and PARK15 linked FBXO7 mutations or variations are summarized in Fig. 1a.

FBXO7 proteins belongs to F-box-containing protein family (FBP) [18, 35]. These FBP proteins possess a F-box domain containing 40 amino acids [41, 42]. FBPs assist in many important cell processes such as formation of protein complexes, maintenance of genome stability, formation of synapse and circadian rhythms [41, 42]. There are three kinds of FBP proteins: FBL (FBP with F-box and leucine-rich-repeat domains), FBW (FBP with F-box and WD40 domains) and FBXO proteins (FBP with F-box domain only) [41, 42]. The FBXO7 belongs to FBXO subfamily and have 5 function domains (from N to C terminus): an UBL domain, a cdk6 domain, a dimerization domain, a F-box domain and a proline rich domain (Fig. 1) [35, 43]. WT FBXO7 proteins have two isoforms, isoform 1 has an UBL domain in its N terminus and is longer than isoform 2 (Fig. 1) [35, 43]. FBXO7 proteins are mainly expressed in cerebral cortex, globus pallidum and SN, and to a lesser extent in hippocampus and cerebellum [44]. Endogenous and overexpressed FBXO7 proteins are mostly localized to cell nucleus [36, 45]. FBXO7 proteins have been found to be positively relevant to tumorigenesis [46]. FBXO7 proteins are reported to be required for the ubiquitination and proteasome-mediated proteolysis of the hepatoma up-regulated protein (HURP), a regulator of mitotic spindle assembly [47]. In　SCF^FBXO7^ complex, FBXO7 recruits HURP through its C-terminal proline-rich region in a Cdk1-cyclin B-phosphorylation dependent manner. Mutation of the multiple Cdk1-cyclin B phosphorylation sites on HURP or the proline-rich region of FBXO7 abolishes the association between FBXO7 and HURP [47]. Furthermore FBXO7 is reported to promote ubiquitination and degradation of cIAP1, an inhibitor of apoptosis family member which regulates canonical and non-canonical NF-κB signalling [48]. Thus FBXO7 may play roles in the regulation of cIAP1 function and cell apoptosis via SCF^FBXO7^ ubiquitin E3 ligase mediated ubiquitination of cIAP1. Recently FBXO7 is found to interact with PI31, a proteasome inhibitor [49]. It is found that there is a dimerization domain in both proteins, which is vital to homodimerization or heterodimerization of FBXO7 and PI31 proteins. However knockdown of FBXO7 does not affect PI31 levels, arguing against PI31 as a genuine substrate for SCF^FBXO7^ E3 ligase [49].

Recent findings demonstrate that FBXO7 is a stress responsive protein and stress of cells can up-regulate FBXO7 expression [26]. The up-regulated expression of WT FBXO7 can protect cells against stress [26]. However PD-linked FBXO7 mutants aggravate neurotoxins induced cell demise [26]. Furthermore stress of cells can promote mitochondria translocation of FBXO7 proteins [26]. WT FBXO7 can facilitate neuroprotective mitophagy, whereas all PD-linked FBXO7 mutations inhibit mitophagy [26]. The mitophagy is an important mitochondrial quality control process and functions to selectively clear away of damaged or impaired mitochondria by autophagy process, which was first mentioned by J.J. Lemasters in 2005 [50]. The mitophagy promotes turnover of mitochondria and prevents accumulation of dysfunctional mitochondria which can lead to neuron degeneration in PD and other human disorders [50]. It is proposed that FBXO7 may participate in mitophagy process via direct interactions with PINK1 and Parkin [51, 52]. FBXO7 may modulate the translocation of Parkin to damaged mitochondria [51]. Overexpression of WT FBXO7, but not PD-linked FBXO7 mutants, can rescue degeneration of DA neurons in Parkin null Drosophila [51]. These findings suggest that FBXO7 may function in a common pathway with Parkin/PINK1 protein pair to modulate mitophagy, whereas FBXO7 mutations lead to loss of these functions.

It is suggested by recent findings that WT FBXO7 protein can have dual protective as well as toxic functions [26]. Stress of cells can up-regulate FBXO7 expression to protect cells. However increased FBXO7 expression under stress can lead to formation of deleterious FBXO7 protein aggregates, especially in mitochondria [26]. PD-linked FBXO7 mutations aggravate toxic FBXO7 aggregation in mitochondria [26]. FBXO7 aggregation in mitochondria can impair mitochondria integrity, promote generation of reactive oxygen species (ROS) and finally lead to cell demise [26]. However FBXO7 aggregation induced proteotoxicity to cells can be alleviated by protein aggregation inhibitors such as L-proline, but be aggravated by prohibitin, a nature mitochondrial protease inhibitor [26]. It is proposed that FBXO7 aggregation, mitochondria impairment and ROS production may form a positive feedback loop implicated in FBXO7-linked neuron degeneration [26]. Such a situation can be aggravated by FBXO7 mutations induced mitophagy inhibition, contributing to PARK15 (Fig. 3) [26].

### Parkin and Parkin-linked PARK2

Parkin mutations is a frequent cause of juvenile-onset Parkinsonism (PARK2) with onset age < 40 years [53, 54]. Parkin mutations have also been implicated in late onset PD [55]. The PARK2 gene is the first autosomal recessive Parkinsonism gene that has been mapped and cloned [13, 16]. PD-Linked Parkin mutations include deletions, multiplications and point mutations in coding regions (Fig. 1b) [56]. Parkin mutations occur in homozygotic, heterozygotic or compound heterozygotic states, mostly in ring domains of Parkin [16, 57]. Several Parkin mutations were identified in Parkin UBL domain and also in unique Parkin domain (UPD) [58, 59]. Parkin mutations induced PARK2 present with rigidity, bradykinesia, dyskinesia, postural instability and dystonia [58–60]. Post-mortem studies revealed that most PARK2 patients did not have Lewy bodies formation except in one case where Lewy bodies were present in SN and locus coeruleus districts [61]. The other cases of PARK2 had severe loss of dopaminergic neurons in SN, Locus coeruleus, and spinocerebellar system [58, 59]. Neurofibrillary tangles can be also identified in cerebral cortex and brainstem nucleus of PARK2 patients [62, 63].

Parkin is a HECT/RING hybrid ubiquitin E3 ligase that physically receives ubiquitin on its catalytic centre and then passes them onto its substrates. The N-terminus of Parkin protein comprises of a UBL and a UPD domains (Fig. 1). The C terminus of Parkin protein consists of two RING fingers domains flanking a in-between RING (IBR) domain (Fig. 1) [57, 62]. The UBL domain is separated from UPD and RING-IBR-RING domains by a linker that comprises of cleavage sites for pro-apoptotic caspases [16, 57]. The UBL domain can bind to subunits of 19S proteasome, which is supposed to modulate proteasome function [62]. 2 RING domains interact with ubiquitin E2 co-enzymes and substrates to execute substrates ubiquitination [62]. Some studies also claim that UBL domain can also bind to substrates [64]. WT Parkin is known to promote cellular proliferation by association with several important cell cycle regulatory molecules and exhibits tumour suppressor effects [21, 56].

Parkin can recognize and interact with its specific targets, execute its E3 ligase activity to promote ubiquitination and proteasomal degradation of its substrates [65, 66]. In Parkin-linked PARK2, abnormal accumulations or deregulation of some potential deleterious Parkin substrates are supposed to be relevant to Parkin mutations induced DA neuron degeneration in PD [20]. WT Parkin, but not PD-linked Parkin mutants, can target and down regulate apoptosis inducing protein Bax to abrogate Bax-induced apoptosis [67]. Thus Parkin mutations may contribute to apoptosis via loss of control on Bax induced toxicity. The Fas-associated factor 1 (FAF1) was a positive modulator for DA neuron degeneration in PD [68]. WT Parkin, but not PD-linked mutant Parkin, can act as an E3 ubiquitin ligase to ubiquitinate and degradate FAF1, thus abrogate FAF1-mediated neuron cell demise [68]. Parkin can also target polyglutamine proteins ataxin-2 and ataxin-3 for ubiquitination to negatively control their levels and reduce these polyglutamine proteins induced cytotoxicity [69, 70]. It is found that α-Sp22 (a 22 KDa glycosylated form of α-syn) can be a substrate of Parkin [71]. However in contrast to WT Parkin, mutant Parkin failed to bind and ubiquitinate α-Sp22 leading to pathological accumulation of α-Sp22 in brain [71]. Other α-syn binding proteins including synphilin-1 and some septins are also found to be substrates of Parkin [72–76]. Co-expression of α-syn, synphilin-1 and Parkin result in the formation of Lewy-body-like ubiquitin-positive cytosolic inclusions [77]. In this case Parkin is suggested to be a dual-function ubiquitin E3 ligase and that K63-linked ubiquitination of synphilin-1 by Parkin may be involved in the formation of Lewy body inclusions associated with PD [75]. Septins are a group of GTP-binding proteins found primarily in eukaryotic cells [78]. Septins have been implicated in the localization of cellular processes at the site of cell division, at the plasma membrane, at sites where specialized structures like cilia or flagella are attached to the cell body [79]. So far two septins (SEPT5/CDCrel-1 and SEPT4/CDCrel-2) are found to be Parkin’s substrates and may potentially play roles in PD pathogenesis [56]. The SEPT4/CDCrel-2 can coaggregate with α-syn as Lewy bodies in PD, and increased level of SEPT5/CDCrel-1 can rapidly eliminate DA neurons in rat heads [75]. Thus WT Parkin may promote down regulation of SEPT4 and SEPT5 to reduce these septins induced adverse impacts on DA neuron viability. Parkin is also reported to target a putative G protein-coupled transmembrane polypeptide, named Pael receptor [80]. This receptor tends to become unfolded, insoluble, and ubiquitinated in vivo and leads to unfolded protein-induced cell death. Parkin specifically ubiquitinates this receptor and promotes the degradation of insoluble Pael receptor to suppress cell degeneration [80]. Parkin is relevant to vesicle formation [81]. The Synaptotagmin XI (important for vesicular trafficking and dynamics) is reported to be a substrate of Parkin [81]. The accumulation of Synaptotagmin XI was found in SN of PD patients [81]. Since synaptotagmin XI is a member of the synaptotagmin family that is well characterized in their importance for vesicle formation and docking, the interaction with this protein suggests a role for Parkin in the regulation of the synaptic vesicle pool and in vesicle release. Loss of Parkin could thus affect multiple proteins controlling vesicle pools, docking and release and explain the deficits in dopaminergic function seen in PD patients with Parkin mutations. Detailed Parkin substrates and their potential implications in PD pathogenesis are discussed in a well-written review paper [56].

On the other hand, Parkin can play important roles in regulating mitochondrial movement, distribution, and clearance [52, 82–86]. Parkin can cooperate with PINK1 to control mitochondria quality via modulation of mitophagy, whereas Parkin mutations impair mitophagy [52, 82, 83]. Upon mitochondria impairment, PINK1 accumulates in defective mitochondria. The accumulated PINK1 can attract Parkin from cytosol and finally cooperatively mediate clearance of damaged mitochondria via mitophagy. Indeed, activation and recruitment of Parkin onto damaged mitochondria involves PINK1-mediated phosphorylation of both Parkin and ubiquitin (Ub). Through a stepwise cascade, Parkin is converted from an autoinhibited enzyme into an active phospho-Ub-dependent E3 ligase. After activation, Parkin can ubiquitinate itself in concert with its many mitochondrial substrates. The PINK1 induced phosphorylation of Ub conjugated to Parkin substrates can trigger further cycles of Parkin recruitment and activation. This feed-forward amplification loop regulates both Parkin activity and mitophagy. It is identified that Parkin mediates the formation of two distinct poly-ubiquitin chains, linked through Lys-63 and Lys-27 [86]. The VDAC1 (voltage-dependent anion channel 1) is recognized as a target for Parkin-mediated Lys-27 poly-ubiquitylation and subsequent mitophagy induction [86]. Besides mitofusion 1 (Mfn1) and mitofusion 2 (Mfn2), large GTPases that mediate mitochondrial fusion, can be substrates of Parkin for mitophagy induction [84]. Parkin is also found to modulate autophagy and mitophagy via mono-ubiquitination of Bcl-2 [87]. Furthermore PINK1 and Parkin are also reported to play role in arresting mitochondrial movement via targeting Miro, a component of the primary motor/adaptor complex that anchors kinesin to the mitochondrial surface [88]. PINK1 phosphorylates Miro and phosphorylation of Miro activates proteasomal degradation of Miro in a Parkin-dependent manner [88]. Removal of Miro from mitochondrion also detaches kinesin from its surface [88]. Thus by preventing mitochondrial movement, the PINK1/Parkin pathway may quarantine damaged mitochondria prior to their clearance via mitophagy [88]. The mitochondrial dynamin related protein-1 (Drp1) can also be the target of Parkin [85]. WT Parkin interacts with and subsequently ubiquitinates Drp1 to promote its proteasome-dependent degradation [85]. Pathogenic mutant Parkin inhibits the ubiquitination and degradation of Drp1, leading to an increased level of Drp1 for mitochondrial fragmentation and cell viability impairment [85].

### Clinical aspects of Parkin and FBXO7 linked PD carriers

Homozygous or heterozygous mutations in Parkin and FBXO7 are both associated with early onset autosomal recessive Parkinsonism (PARK2 and PARK15). The common clinical features of PARK2 and PARK15 include classic PD symptoms including rigidity, bradykinesia and postural instability [89, 90]. In addition they have atypical features such as hyperreflexia, foot dystonia, sleep benefit, diurnal fluctuations and early susceptibility to levodopa induced dyskinesia [91]. Compared with PARK2, the FBXO7-linked PARK15 progress more rapidly and is more severe. In PARK 15, Parkinsonism can be accompanied with signs of cognitive decline, loss of sustained levodopa responsiveness, severe levodopa induced bradykinesia as well as pyramidal signs such as hyperreflexia and babinski signs [15, 17]. PARK15 carriers also exhibit other signs such as eyelid aphaxia, slow saccade and early imbalance in posture [92]. However resting tremor is not a common feature of PARK 15 [44]. We have analyzed and summarized the onset age of PARK2 and PARK15 based on published reports [17, 18, 20–22, 51, 52, 54, 57, 58, 74–76, 78, 89]. About 38 cases of PARK2 and 9 cases of PARK15 patients with detailed onset ages were analyzed (Table 1). After ANOVA statistic analysis, we find that PARK15 has significant earlier PD onset age than PARK2 (Table 1). Furthermore it appears that PARK15 has a wider and more complicated network of neuronal degeneration than PARK2 [36, 44].

**Table 1:** Onset ages of PARK2 and PARK15

PARK2 [15, 55, 58, 94–97]		PARK15 [17, 18, 44, 45, 92]	
Case no	Ages	Case no	Ages
1	7	1	10
2	10	2	13
3	13	3	17
4	13	4	17
5	13	5	18
6	15	6	19
7	15	7	20
8	16	8	22
9	18	9	24
10	18		
11	19		
12	20		
13	20		
14	22		
15	23		
16	24		
17	26		
18	27		
19	28		
20	28		
21	28		
22	28		
23	29		
24	29		
25	29		
26	30		
27	30		
28	31		
29	32		
30	33		
31	34		
32	34		
33	45		
34	45		
35	47		
36	54		
37	61		
38	65		
Average ± SD	27.87 ± 13.37	17.78 ± 4.3*	

*P = 0.032

### Pathophysiologic aspects of Parkin and FBXO7 linked PD

**Fig. 2:**
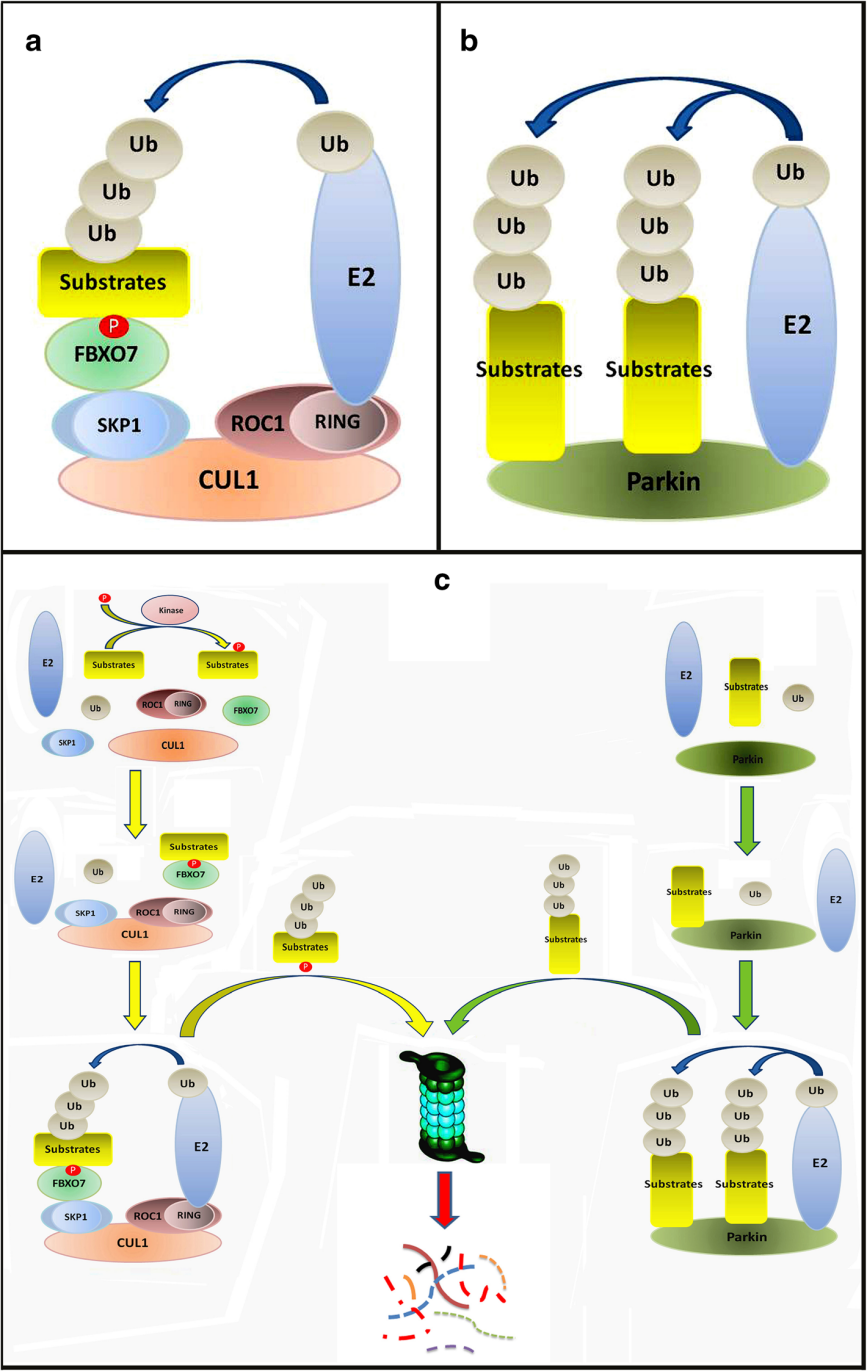
Physiological roles of FBXO7 and Parkin in UPS. **a**, the physiological roles of FBXO7 in SCF^FBXO7^ E3 ligase complex. FBXO7 acts as an adaptor protein to recognize and interact with its substrates for SCF^FBXO7^ E3 ligase mediated ubiquitination. After interaction with its substrates, FBXO7 will bind with SKP1 which further interact with Cul1 and ROC1 to form functional SCF^FBXO7^ E3 ligase complex. The SCF^FBXO7^ E3 ligase will mediate E2 ligase induced ubiquitination of FBXO7 substrates. **b**, the physiological roles of Parkin. After interaction with its substrates, Parkin exerts its E3 ligase activity to mediate ubiquitination of its substrates by E2 ligase. **c**, detailed procedures of SCF^FBXO7^ and Parkin E3 ligases mediated ubiquitination and proteasome degradation of substrates. (*Left*), SCF^FBXO7^ E3 ligase mediated substrates recognition, ubiquitination and proteasomal degradation. (*Right*), Parkin mediated substrates recognition, ubiquitination and proteasomal degradation

There are overlapping and divergent pathophysiological roles between Parkin and FBXO7. The physiological roles of Parkin and FBXO7 are summarized in Fig. 2. Both Parkin and FBXO7 play a common role in UPS [35, 67, 98]. The Parkin is a HECT/RING hybrid ligase, whereas FBXO7 is an adaptor protein in Skp-Cullin-F-box (SCF) SCF^FBXO7^ ubiquitin E3 ligase complex [12, 43]. Parkin and FBXO7 are both implicated in tumorigenesis [51]. WT Parkin and FBXO7 can both be cytoprotective, whereas their PD-linked mutants are all deleterious to DA neurons. Both WT Parkin and FBXO7 promote mitophagy, whereas their PD-linked mutants impair mitophagy. Furthermore FBXO7 and Parkin have reciprocal interactions, suggesting overlaps of their signalling pathways. WT FBXO7 but not PD-linked FBXO7 mutants can rescue lack of Parkin induced PD symptoms in flies [51]. FBXO7 was reported to mediate mitochondria translocation of Parkin [51]. Therefore FBXO7 may act upstream of Parkin and have Parkin independent complicated functions. Our recently findings show that FBXO7 proteins can form deleterious aggregates in mitochondria [26]. However no reports have shown that Parkin can form protein aggregates in mitochondria. The similarities and differences of their pathophysiological roles are summarized in Table 2.

**Table 2:**
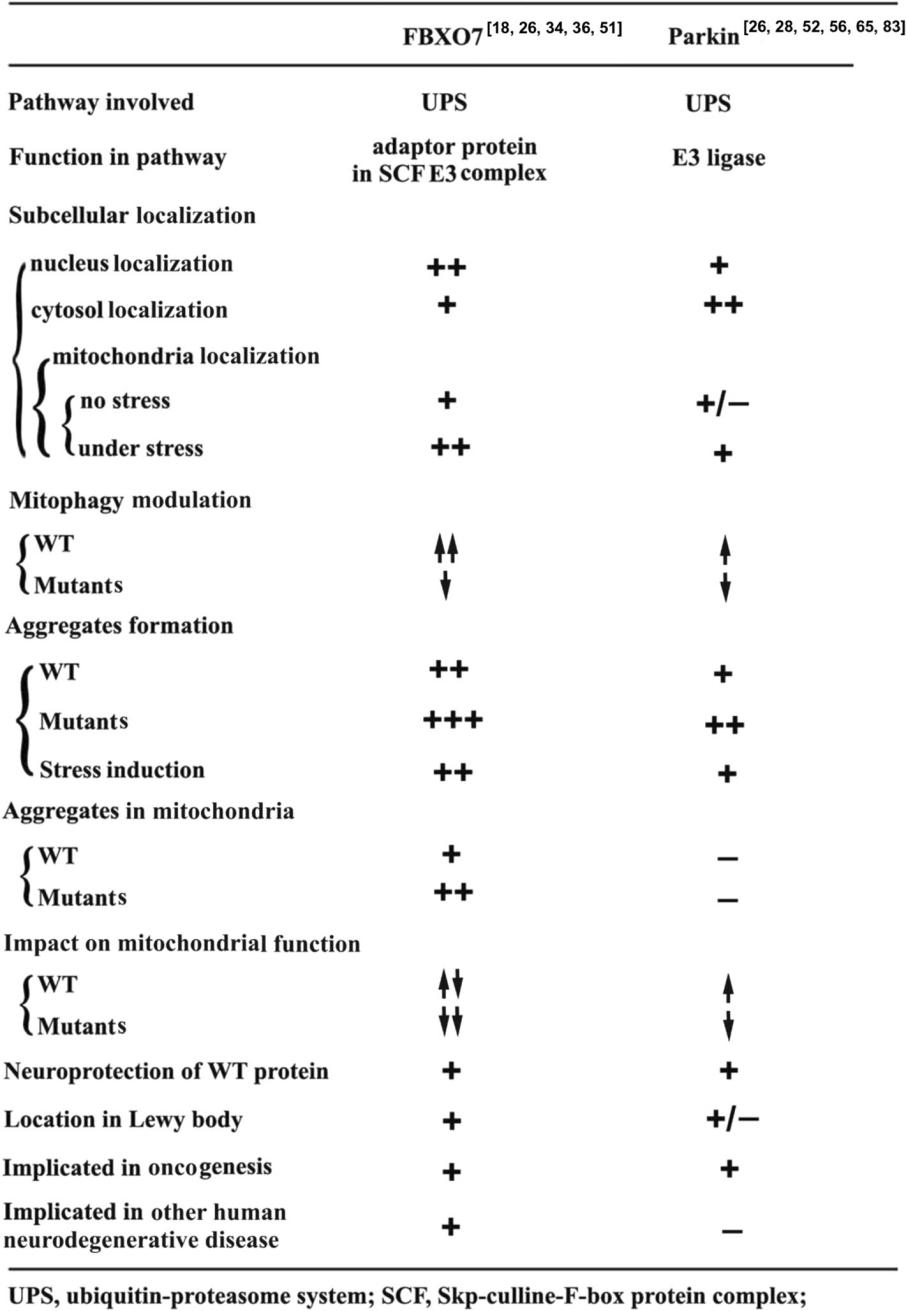
Comparison of physiological and pathological features of FBXO7 and Parkin

## Conclusions

**Fig. 3:**
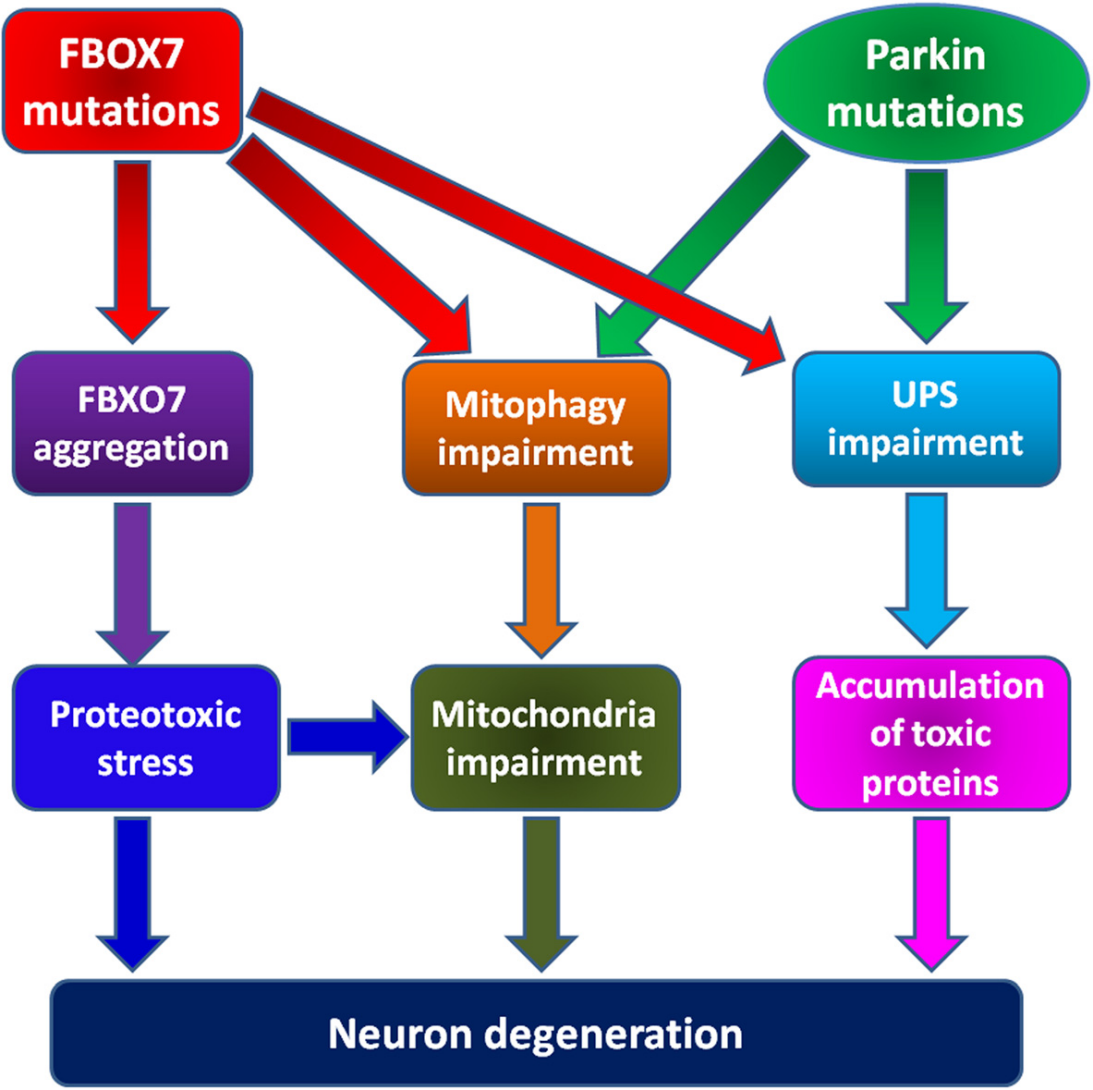
Potential pathogenesis of FBXO7 and Parkin mutations induced neuron degeneration in PD. FBXO7 mutations can lead to deleterious FBXO7 protein aggregation, inhibition of mitophagy process and impairment of FBXO7-linked UPS functions. Mutant FBXO7 proteins can form stress dependent toxic protein aggregates in mitochondria. The impaired mitophagy will also impair mitochondria functions. Besides, the impairment of FBXO7-linked UPS function may lead to accumulation of some toxic FBXO7 targets. All these alterations may converge and contribute to FBXO7 mutations induced neuron degeneration in PARK15. However Parkin mutations induced mitophagy impairment and accumulation of toxic Parkin targets may contribute to neuron degeneration in PARK2

Mutations of Parkin and FBXO7, two proteins in UPS, are both implicated in pathogenesis of early onset DA neuron degeneration in PD (PARK2 and PARK15). Both WT Parkin and FBXO7 can facilitate, but their PD-linked mutants impair neuroprotective mitophagy. However WT FBXO7 can have dual protective or deleterious functions. FBXO7 proteins, especially PD-linked FBXO7 mutants, can form deleterious protein aggregates in mitochondria under stress, leading to proteotoxic stress in mitochondria. Taken together, FBXO7 mutations may lead to neurodegeneration via impairment of UPS, inhibition of mitophagy and formation of deleterious FBXO7 aggregates. On the other hand Parkin mutations may induce neuron degeneration via impairment of UPS together with inhibition of mitophagy (Fig. 3). The pathophysiologic variances between FBXO7 and Parkin can partially account for different clinical phenotypes of PARK15 and PARK2 (Fig. 3). Further studies are needed to unravel the common pathophysiologic links between these two proteins with the hope to identify specific pathways for targeted therapy in PD.

### Ethics approval

Not applicable, as no human subjects have been directly involved.

### Availability of data and materials

Not applicable, as no original data has been used in the current review manuscript.

## Abbreviations

AD: Alzheimer’s disease
CDCrel: cell division control related protein
DA: dopamine
Drp1: dynamin related protein-1
FAF1: Fas-associated factor 1
FBP: F-box-containing protein
FBXO7: F-box protien 7
FPD: familial form Parkinson’s disease
HSP90: heat shock protein 90
IBR: in-between RING domain
LRRK2: Leucine rich repeat kinase 2
mtUPR: mitochondria unfold protein response
PD: Parkinson’s disease
ROS: reactive oxygen species
SCF: Skp-Cullin-F-box E3 ligase complex
SN: substansia nigra pars compacta
SPD: sporadic Parkinson’s disease
UBL domain: ubiquitin like domain
UPD: unique Parkin domain
UPS: ubiquitin proteasome system
WT: wild type
α-syn: α-synuclein
